# Astrocytes in Alzheimer’s Disease: Pathological Significance and Molecular Pathways

**DOI:** 10.3390/cells10030540

**Published:** 2021-03-04

**Authors:** Pranav Preman, Maria Alfonso-Triguero, Elena Alberdi, Alexei Verkhratsky, Amaia M. Arranz

**Affiliations:** 1 VIB Center for Brain & Disease Research, 3000 Leuven, Belgium; pranav.preman@kuleuven.be; 2 Laboratory for the Research of Neurodegenerative Diseases, Department of Neurosciences, Leuven Brain Institute (LBI), KU Leuven (University of Leuven), 3000 Leuven, Belgium; 3 Achucarro Basque Center for Neuroscience, 48940 Leioa, Spain; maria.alfonso@achucarro.org (M.A.-T.); elena.alberdi@ehu.es (E.A.); 4 Department of Neurosciences, Universidad del País Vasco (UPV/EHU), 48940 Leioa, Spain; 5 Centro de Investigación Biomédica en Red de Enfermedades Neurodegenerativas (CIBERNED), 48940 Leioa, Spain; 6 Faculty of Biology, Medicine and Health, University of Manchester, Manchester M13 9PT, UK; 7 Ikerbasque Basque Foundation for Science, 48009 Bilbao, Spain

**Keywords:** astrocyte, Alzheimer´s disease, neurodegeneration, transcriptomics, RNA sequencing (RNA-seq), cellular states

## Abstract

Astrocytes perform a wide variety of essential functions defining normal operation of the nervous system and are active contributors to the pathogenesis of neurodegenerative disorders such as Alzheimer’s among others. Recent data provide compelling evidence that distinct astrocyte states are associated with specific stages of Alzheimer´s disease. The advent of transcriptomics technologies enables rapid progress in the characterisation of such pathological astrocyte states. In this review, we provide an overview of the origin, main functions, molecular and morphological features of astrocytes in physiological as well as pathological conditions related to Alzheimer´s disease. We will also explore the main roles of astrocytes in the pathogenesis of Alzheimer´s disease and summarize main transcriptional changes and altered molecular pathways observed in astrocytes during the course of the disease.

## 1. Introduction

In 1856, Rudolf Virchow introduced the concept of neuroglia as a connective tissue of the brain and the spinal cord that holds together nervous elements [1]. Glial cells have been in focus of research of many prominent neuroanatomists of the 19th century; in particular morphology of parenchymal glia characterized by stellate appearance when stained by Golgi technique has been minutely characterised [2]. These stellate cells received the name of astrocytes (αστρονκψτοσ; *astron, star and kytos, a hollow vessel,* later *cell* i.e., star-like cell) [3]. Rather prophetically, Lenhossék proposed to call all parenchymal glial cells “spongiocytes” and he only named a subpopulation of them as astrocytes. Astrocytes belong to the class of neural cells known as astroglia, which covers several different cell types including astrocytes proper, radial astrocytes, velate astrocytes, tanycytes, pituicytes, ependymocytes, choroid plexus cells and retinal pigment epithelial cells. Astrocytes are parenchymal homeostatic and defensive cells of the central nervous system (CNS). Recent data provide clear evidence that astrocytes actively contribute to the pathogenesis of neurodegenerative disorders, with particular role in Alzheimer’s disease, Parkinson disease, Huntington disease, multiple sclerosis and amyotrophic lateral sclerosis. In this review, we provide overview of the multifaceted roles of astrocytes in physiological as well as pathological conditions related to Alzheimer´s disease. We also explore mechanisms by which astrocytes contribute to Alzheimer´s and summarize main transcriptional changes and altered molecular pathways observed in astrocytes during the course of Alzheimer´s disease.

## 2. Astrocytes in the Healthy Brain

### 2.1. Origin, Development and Numbers

#### 2.1.1. Origin

**Figure 1:**
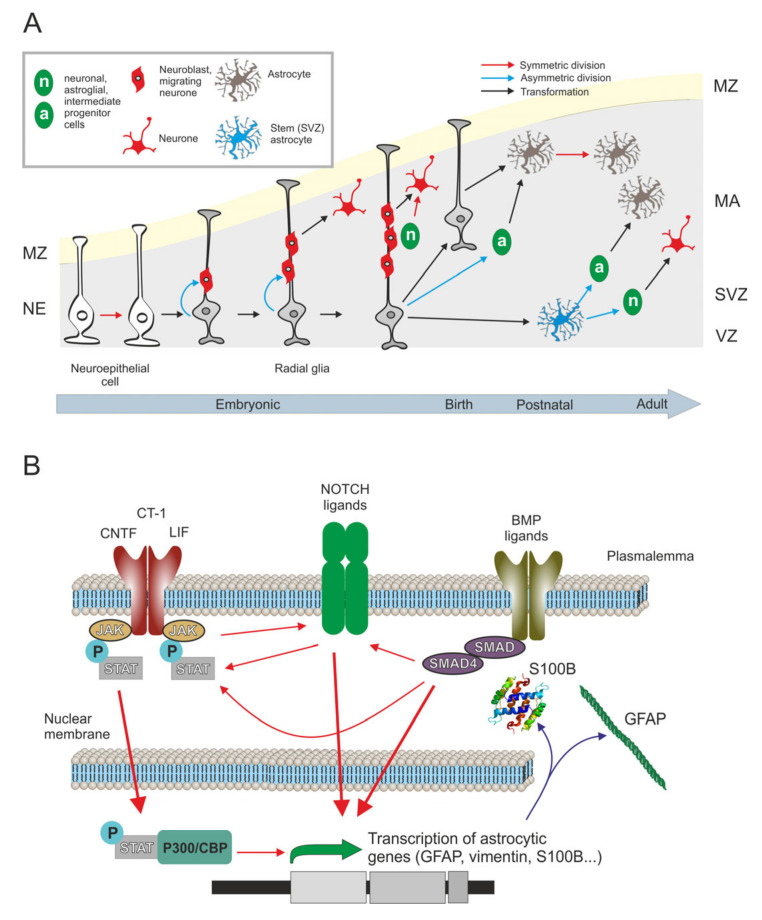
Origin and development of human astrocytes in the healthy brain. (**A**) Astrocytes originate at pre- and post-natal stages by symmetric or asymmetric division as well as by direct transformation of radial glia, intermediate glial progenitors, already differentiated astrocytes and NG2 glia. (**B**) Three signalling pathways determine astrocytic development: the JAK/STAT (Janus Kinases and signal transducer and activator of transcription proteins), NOTCH (Notch homolog 1) and BMP-SMAD (Bone Morphogenetic Proteins and “Small Mothers Against Decapentaplegic”). These pathways act synergistically allowing the transcription of astroglial genes. MZ: marginal zone, MA: mantle, SVZ: subventricular zone, VZ: ventricular zone.

Astrocytes, similar to neurones and oligodendroglia, originate from neuroepithelium-derived radial glial cells. At prenatal stages, astroglial precursors are produced by asymmetric division of radial glial cells. The bulk of astrocytes however emerges postnatally and the major source for astrogenesis is associated with symmetric division of differentiated astrocytes; this division was initially described by Ramon y Cajal in a form of twin astrocytes or “*astrocitos gemelos*” [4]. Astrocytes can also emerge from direct transformation of radial glia or differentiate from NG2 glial cells also known as oligodendrocyte precursors or OPCs (Figure 1A) [5,6]. Intermediate glial progenitor cells, originated from asymmetric division of radial glia, generate immature astrocytes that migrate towards the cortical layers and proliferate through symmetric division. In layer I of the embryonic and neonatal cortex there are other type of neural progenitors that give rise to the astrocytes of superficial layers (I–IV) (Figure 1A) [7].

#### 2.1.2. Prenatal Astrogenesis

In foetal brain development, gliogenesis follows neurogenesis. Molecular mechanisms that govern differentiation of astrocytes are mainly determined by the expression of two astrocytic genes: intermediate filament glial fibrillary acidic protein (GFAP) and calcium binding protein (S100β) [8,9]. Three signalling pathways, JAK-STAT, Notch and BMP-SMAD, determine the embryonic development of astrocytes. The IL-6 family of cytokines (CNTF, LIF, CT-1) are primarily responsible for initiating gliogenesis [10]. This family activates the canonical **JAK/STAT signalling pathway**; activated STAT is responsible, together with the p300/CBP co-activator complex, for promoting transcription of astroglial genes to instigate formation of astrocytes [9,11,12,13] (Figure 1B). In the course of astrogenesis, JAK/STAT and **Notch signalling** pathways act synergistically: activation of JAK produces the release of Notch ligands to activate this pathway; Notch activity, on the other hand, induces the phosphorylation of STAT thus activating JAK/STAT cascade [12] (Figure 1B). Notch is also involved in the demethylation and, therefore, in epigenetic regulation of astrocytic genes during differentiation. In neurogenesis, the promoter of the astrocytic gene glial fibrillary acidic protein (GFAP) is epigenetically silenced through methylation by DNA methyltransferase I (DNMT1). When astrogenesis begins, Notch signalling pathway activates DNMT1 release, allowing GFAP transcription and astrogenesis. Epigenetic regulation of astrocytic genes is also regulated by JAK/STAT pathway since acetylation of histones by p300/CBP enhances the opening of chromatin [12]. Notch cascade also promotes astrogenesis by directly activating the GFAP promoter [8]. In addition, **BMP ligands**, members of the transforming growth factor beta (TGF-β) signalling ligands superfamily, bind to and activate their respective receptors inducing SMAD phosphorylation and its dimerisation with SMAD4. The SMAD-SMAD4 complex is a transcriptional activator of astrocytic genes such as GFAP and calcium-binding protein β (S100β) which promote astrogenesis (Figure 1B). This astrogenesis signalling pathway has been described in progenitor cultures at embryonic day 14 and later; besides promoting astrogenesis this pathway suppresses neuronal and oligodendrocytic differentiation [10,14,15,16]. Both JAK-STAT and Notch pathways are also activated by BMP signalling [10,17].

#### 2.1.3. Postnatal Astrogenesis

The second, and the largest wave of astrogenesis occurs postnatally. During postnatal astrogenesis, approximately 50% of all astrocytes are generated from the symmetric division of differentiated astrocytes [18](Figure 1A). In this second wave, protoplasmic astrocytes are also generated by direct transformation of radial glia, which lose their apical processes; besides, astrocytes can arise from NG2 glial cells [19,20,21] (Figure 1A). The importance of the BMP-SMAD signalling pathway in adult astrogenesis is well documented: inactivation of this pathway reduces the expression of astrocytic genes such as GFAP and S100β, and decreases the number of astrocytes [16]. In contrast, the number of GFAP-positive astrocytes increases substantially in a mouse model overexpressing BMP [22]. More studies are needed to elucidate other potential signalling cascades involved in postnatal astrogenesis.

#### 2.1.4. Astrocyte Numbers

There is some controversy about the total number of astrocytes, and their proportion in different brain regions remains to be elucidated. Isotropic fractionation and quantitative unbiased stereology estimate that all glia accounts for ~40% of all cells in the human brain; the ratio of non-neuronal cells to neurones varies depending on the region [23,24,25] being 0.2:1 in the cerebellum, 3.7:1 in the cortex and up 7:1 in the spinal cord and 11:1 in the brain stem [7].

In different brain regions astrocytes account for 20–40% of the total glial population, suggesting that oligodendrocytes are slightly more numerous [7,23]. Stereology studies (without immunocytochemistry) on post-mortem human brain samples report 75% oligodendrocytes, 20% astrocytes and 5% microglia in neocortex [26]. In mouse cortex the ratio of astrocytes to neurones is around 0.2 [27].

### 2.2. Astrocyte Functions in Healthy Brain

Astrocytes perform a wide variety of critical functions determining normal operation of the nervous tissue. Numerous receptors expressed in astrocytes allow them to sense neuronal activity [28], activation of these receptors trigger astrocytic ionic signalling, mainly mediated by changes in cytosolic concentration of Ca^2+^ and Na^+^ [29] which control a multitude of plasmalemmal “homeostatic” transporters [30]. In addition, astrocytic excitability is supported by second messengers such as ATP [31]. These transporters are responsible for K^+^ buffering, clearance of neurotransmitters including glutamate, ATP, GABA, adenosine and endocannabinoids among others, maintaining synaptic transmission, preventing excitotoxicity and providing for neuroprotection [7,32]. These transporters specifically concentrate in distal astroglial processes that enwrap synaptic contacts; the perisynaptic membranous sheath form the astroglial cradle, essential for all aspects of synaptic function from synaptogenesis and synaptic maintenance to synaptic extinction [33]. Astrocytes promote synaptogenesis by producing and secreting critically important factors such as cholesterol, glypicans, hevin and thrombospondins [34,35]. They also control synapse elimination by direct phagocytosis [36] or by modulating microglia synaptic pruning in a complement dependent process [37]. Astrocytic endfeet contact blood vessels and, together with endothelial cells and pericytes, create the blood-brain barrier (BBB) which separates the highly controlled brain microenvironment from the peripheral blood circulation [38]. Astrocytes form a functional and anatomical link between the vasculature and the CNS parenchyma through the neurogliovascular unit [39], regulate local blood flow and contribute to energy supply in the form of lactate to neurones [40,41]. They store glycogen, which is metabolised to pyruvate and lactate, with the latter transported across the cell membrane and delivered to neighbouring neurones. Astrocytes are fundamental for operation of the glymphatic system, an organised pathway for elimination of soluble proteins, waste products, and excess extracellular fluid from the brain, in which clearance is facilitated by astrocytic aquaporin 4 (AQP4) water channels [42,43]. Finally, they control extracellular space volume and are also in charge of the homeostatic maintenance of the CNS by transporting extracellular ions, protons and metabolites, and controlling levels of pH and water [7].

### 2.3. Astrocyte Diversity

Although belonging to the same class of neural cells and sharing same basic properties (such as high K^+^ permeability, expression of transporters providing for molecular homeostasis, ionic excitability, etcetera) [7], there is a prominent inter- and intra-regional heterogeneity among astrocytic populations at both morphological and molecular levels, which translates into differential functional properties. Heterogeneity of astrocytes might be explained, at least in part, by their diverse place of birth and association to specific type of progenitors. Intrinsic programs within the astrocytic precursors and extrinsic signals from neighbouring cells can also influence the diversity.

#### 2.3.1. Morphological Subtypes of Cortical Astrocytes: Protoplasmic, Interlaminar, Varicose-Projection and Fibrous Astrocytes

There are four main morphologically distinct subtypes of astrocytes in the human neocortex while only two have been found in rodents:

*Protoplasmic astrocytes* represent the most abundant type of astroglia in the grey matter and are located in cortical layers II to VI [44]. They are characterised by a small cell body, of approx. 10 µm in diameter with many large processes (up to 40 in humans, several in rodents). These processes extend radially from the soma, and many complex and fine lateral branches are born from them, defining the territory of astrocyte domain. Territorial domains of cortical protoplasmic astrocytes show very little (<5%) overlap [45]. The volume of human protoplasmic astrocytes is about 10 to 20 times greater than that of rodent astrocytes [46]. 

**Figure 2:**
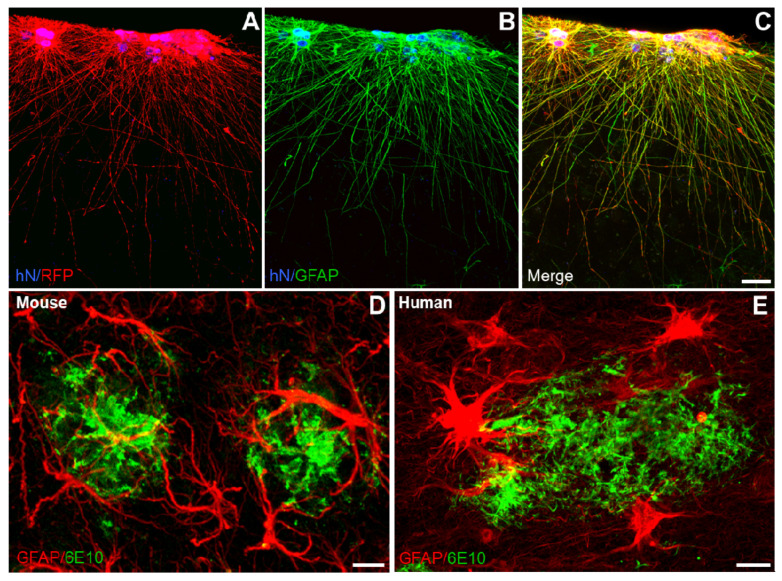
Astrocyte morphologies in healthy and in Alzheimer’s disease brains. (**A**–**C**) Human iPSC-derived astroglial progenitors transplanted into the mouse brain (RFP, red) integrate in the cortex and develop into interlaminar astrocytes expressing GFAP (green). hN: human Nuclei stains the nuclei of human cells. Scale bar: 25 µm. (**D**–**E**) Close interaction of both mouse and human astrocytes with β-amyloid plaques. GFAP-positive mouse or human astrocytes (red) around β-amyloid plaques (6E10, green) in the cortex of an APP/PS1 mouse (**D**) and in the entorhinal cortex of an Alzheimer´s disease patient brain (**E**). Scale bars: 10 µm.

*Interlaminar astrocytes* are almost exclusively found in higher primates (the processes of the so called "rudimentary interlaminar astrocytes" described in mouse [47] do not cross the Lamina 1 and hence cannot be defined as interlaminar), and emerge at postnatal stages. Their somata are located in layer I of the cerebral cortex. These cell bodies are around 10 µm diameter; and several generally unbranched processes emanate from them. These processes are of two types: shorter fibres directed towards the cortex surface that contribute to the astrocytic network underneath the pia mater, and very long fibres that penetrate through the deep layers of the cortex (layers III–IV). Interlaminar astrocytes do not occupy specific territorial domains and overlap with their neighbours. They express markers of radial glia (Pax6, Sox2, and Nestin), as well as astrocytic markers GFAP, S100β, Aqp4, and GLAST [47]. Grafting human iPSC-derived astrocyte progenitors in the mouse brain results in appearance of GFAP-positive interlaminar astrocytes in layer I of the mouse cortex (Figure 2A). Although functions of interlaminar astrocytes remain enigmatic, their structure suggests an essential role in intra-cortical communication [44,46,48,49].

*Varicose-projection astrocytes* are similarly found only in primate brains. These cells are located in cortical layers V to VI. Their numbers are low and they strongly express GFAP. They have several short and straight processes as well as one to five very long (up to 1 mm) processes that are usually straight, unbranched, and have numerous beads or varicosities distributed about 10 μm apart. Unlike protoplasmic astrocytes, they are not organised into well-defined spatial domains and their processes cross through domains of neighbouring astrocytes. Their functions are unclear, arguably varicose-projection astrocytes contribute to long-distance communication through cortical layers and even between grey and white matter [44,46,49]. 

*Fibrous astrocytes* reside in white matter tracts; human astrocytes are much larger than rodent ones. Fibrous astrocytes have a small round soma and straight nonbranched processes. Their fibres overlap, but their bodies do not; they are equidistant from each other. Their processes extend multiple finger-like cytoplasmic protrusions that are directed into the perinodal spaces of the surrounding axons. In addition, fibrous astrocytes contact blood vessels through their processes and endfeet, as do protoplasmic astrocytes [44,46,50]. 

While GFAP has proved to be a reliable marker of astrocytes in vitro, not all astrocytes are immunopositive for GFAP in physiological conditions. Regional differences are also reported with higher GFAP expression in hippocampal than in cortical, striatal or thalamic astrocytes [51]. For reliable characterisation of astrocytic subtypes, immunohistochemical morphometry must utilise additional markers, including cytosolic (such as S100β, glutamine synthetase (GS), aldolase C, ALDH1L1) that allow a better visualisation of the morphological profiles. Astroglia-specific fluorescent reporter mice (i.e., ALDH1L1-GFP), astroglia-specific Cre lines or intraglial injection of fluorescent dyes can also improve morphological characterization [52,53].

#### 2.3.2. Molecular Diversity and Functional Implications

The outbreak of new sequencing methodologies provides for remarkable expansion of our knowledge of molecular diversity of astrocytes. Specialised subpopulations of astrocytes have been recently identified across different brain regions by RNA sequencing in astrocyte-specific reporter mice [54,55,56]. While all astrocytes are strongly enriched in pan-glial gene signatures, each subpopulation shows a unique molecular profile across regions. Distinct sub-populations of astrocytes also exhibit differences in morphology, electrophysiology and calcium signalling [54,56]. These sub-populations also differ in migratory and proliferative capacities, synaptic coverage and ability to support synaptogenesis and neuronal growth and maturation [54,55,56], further corroborating astrocyte diversity tailored to support specific brain regions. Even within a specific brain region, such as cortex, astrocytes in different layers show distinct morphological features, gene signatures, functional properties and cell-surface markers [57,58], indicating the high adaptive potential of these cells.

Between and within-regional astrocyte diversity has recently been confirmed by single-cell RNA sequencing and in situ analyses. Molecularly distinct astrocytic subtypes have been described within the cortex, identifying superficial, mid and deep layer astrocyte gene profiles in a layer patterning that differs from those of neurones [59]. Moreover, up to five molecularly distinct astrocyte subtypes have been identified in mouse cortex and hippocampus, each showing specific morphologies and distinct Ca^2+^ dynamics [60], further highlighting region-dependent functional diversity.

In summary, astrocyte gene expression varies between as well as within brain regions, with astrocytes from each individual brain area showing a subtle and specific gene expression gradient. These molecular differences correlate with distinct morphological features both having functional implications that are beginning to emerge.

## 3. Astrocytes in Alzheimer’s Disease

### 3.1. Major Roles of Astrocytes in Alzheimer´s Disease

Alzheimer’s disease (AD) is characterised by amyloid-β accumulation (β-amyloid or senile plaques), formation of hyperphosphorylated tau neurofibrillary tangles, neuroinflammation, synaptic demise, neuronal death and brain dysfunction leading to severe cognitive impairment. The amyloid hypothesis originally postulated a linearity of progression according to β-amyloid accumulation, which subsequently leaded to formation of tangles and other pathological hallmarks [61]. More recent observations demonstrated that such linear model needs to consider the contribution of different brain cells [62]. Evolution of AD takes long time, with brain defences sustaining homeostasis for decades before cognitive disability becomes apparent in advanced stages of the disease [62]. This cellular defensive phase represents the biological equivalent of preclinical AD [63] and involves complex circular and parallel pathways and poorly characterised homeostatic responses associated with different types of brain cells [64].

The role for glial cells, and for astrocytes in particular, in neuropathology of many neurodegenerative diseases is universally acknowledged [28,65]. The risk of AD is associated with genes mainly expressed by glial cells, either astrocytes, microglia and/or oligodendrocytes [66]. Apolipoprotein E (*APOE*), a major genetic risk factor in Late-Onset AD (LOAD), is mainly expressed in astrocytes in the healthy brain [67] and contributes to accumulation of β-amyloid in the brain [68,69]. Other genes associated with AD such as Clusterin (*CLU*) and Fermitin family member 2 (*FERMT2*) are similarly predominantly expressed by astrocytes. Reactive astrogliosis is prominent in AD being an early event in human patients and in animal models, possibly even preceding the formation of β-amyloid plaques [70,71,72]. These data suggest a crucial role of astrocytes in the pathogenesis of AD.

**Figure 3:**
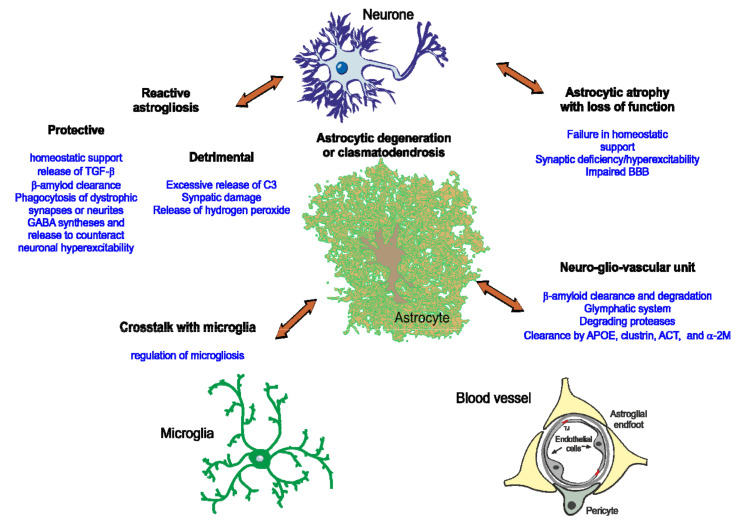
Contribution of astrocytes to Alzheimer´s disease. During the course of AD, astrocytes interact with neurones, microglia and other CNS cells by releasing feed-forward signals and contributing to the vicious cycle leading to neurodegeneration. While reactive astrocytes potentially have both protective and detrimental functions during the course of AD, atrophic astrocytes might lose their homeostatic functions. Astrocyte contribution to β-amyloid degradation and clearance will also influence AD progression.

Morphological studies in post-mortem AD patient brains demonstrated close interaction between astrocytes and β-amyloid depositions [73] (Figure 2B). It is however unclear how this close interaction translates into the disease progression. Astrocytes, when associated with senile plaques, become reactive with morphological hypertrophy manifested by thicker processes and increased expression of the intermediate filament proteins glial fibrillary acidic protein (GFAP), vimentin, nestin and synemin [74]. Reactive astrocytes are found in both human AD patient brains [75] and AD mice models (Figure 2B) [65,76,77]. Pathological signals inducing astrogliosis in AD can be associated with damaged cells; β-amyloid by itself is a strong instigator of astrocyte reactivity. At molecular level, β-amyloid induction of astrogliotic remodelling is mediated by Ca^2+^ release from the endoplasmic reticulum; inhibition of the latter suppresses astrocytic reactivity [78]. In AD, astrocytes undergo relatively mild isomorphic gliosis and astrocytic domains do not overlap, potentially indicating a defensive nature of the astrocytic response. Indeed, inhibition of astrogliosis exacerbates β-amyloid accumulation and histopathology in AD mice [79]. Reactive astrocytes in the vicinity of plaques display aberrant calcium dynamics [80,81]. Astrocyte Ca^2+^ hyperactivity could promote the release of detrimental factors, alter neurone-glia communication and impair synaptic transmission and plasticity [82,83] (Figure 3).

Besides substantial astroglial reactivity, atrophic astrocytes are also present in post-mortem brains of AD patients [48,84] and mouse models of AD [65]. In particular, human AD brains are characterised by severe disruption or even complete disappearance of interlaminar astrocytes [48]. Atrophic astrocytes are characterised by reduced volume and thinner processes as revealed by morphometric analysis of cells immunolabelled with antibodies against GFAP, S100β [85] and GS [86]. In the 3xTg-AD mice model, atrophic astrocytes appear as early as 1-month age in the entorhinal cortex (EC) and the atrophy is sustained after 12 months of age when β-amyloid plaques begin to appear [85]. Similar astroglial atrophy has been described in other models of AD including 5xTG-AD mice, PDAPP-J20 transgenic mice and Swiss 3 [87,88,89,90]. Human astrocytes derived from induced pluripotent stem cells (iPSC) from patients with both familial and sporadic forms of AD also show atrophic phenotypes in vitro compared to control cells [91]. While atrophy might lead to loss of astrocyte homeostatic functions and give rise to synaptic dysfunction, increased excitability and/or damage of the BBB, (Figure 3) very little functional data are available. Finally, neurodegenerative process may directly damage astrocytes resulting in clasmatodendrosis, characterised by fragmentation and disappearance of distal fine processes, along with swelling and vacuolation of the cell body [92] (Figure 3).

Astrocytes could be, in principle, involved in β-amyloid production as they upregulate β-secretase 1 and the amyloid precursor protein (APP) in the diseased brain [82], however no quantitative data points to astrocytes as the major source of β-amyloid. Astrocytes more likely participate in β-amyloid clearance and elimination by different mechanisms. Astrocytes express aquaporin 4 (AQP4) water channels in their vascular end-feet and play an essential role in the glymphatic system implicated in the clearance of β-amyloid [42,43] (Figure 3). They also produce β-amyloid degrading proteases that cleave the peptide into smaller fragments. The metalloendopeptidases neprilysin (NEP), insulin-degrading enzyme (IDE), and endothelin-converting enzymes 1 and 2 (ECE1 and ECE2) are all expressed in astrocytes and contribute to the degradation of monomeric β-amyloid species [93]. Astrocytes also express matrix metalloproteinases MMP-2 and MMP-9 which degrade both fibrillar and monomeric β-amyloid [93] (Figure 3). Clearance of β-amyloid can be mediated by extracellular proteins APOE, ApoJ/Clusterin, α1-antichymotrypsin (ACT) and α2-macroglobulin (α2-M), all produced by astrocytes (Figure 3); these proteins promote the transport of β-amyloid across the BBB to the circulation either alone or in association with LRP1 and VLDLR receptors [93]. Recent studies report that iPSC-derived human astrocytes and mouse astrocytes expressing APOE4 are less efficient in clearing β-amyloid than those expressing APOE3 [94,95]. In addition to β-amyloid clearance, APOE also regulates β-amyloid seeding with APOE4 more potently affecting seed formation than APOE3. APOE affects plaque size and neuritic dystrophy without having much influence on total amyloid load [96,97]. Expression of APOE4 also leads to degeneration of pericytes thus facilitating breakdown of the BBB further contributing to cognitive impairment in APOE4 carriers [98]. 

In AD, reactive astrocytes interact with neurones, microglia and oligodendrocytes by releasing feed-forward signals and contributing to the vicious cycle that leads to neurodegeneration. Of note, β-amyloid can activate the NF-κB pathway in astrocytes, which leads to release of the complement protein C3 (Figure 3). The C3 binding to the microglial receptor C3aR alters β-amyloid phagocytosis while the C3 binding to the neuronal receptor C3aR disrupts dendritic morphology and network function, both effects contributing to AD pathogenesis [99]. Both NF-κB and C3 cascades are activated in human AD brain and in AD mouse models [100,101]. Microglia can also activate astrocytes by secreting specific cytokines (IL-1α, TNFα, and C1q) [101]. This type of reactive astrocytes upregulate classical complement cascade genes including C3 and lose ability to promote synapse formation and function, and to phagocytose synapses and myelin debris [101]. About 60% of the astrocytes in the prefrontal cortex of AD patients are C3-expressing astrocytes [101] and could contribute to neuronal damage; although further analyses are needed for confirmation. In AD, reactive astrocytes participate in shifting the excitation-inhibition balance through secretions of GABA. In the healthy brain, astrocytes do not contribute much to GABA production, however, in AD GABA starts to be synthesised by astrocytes through putrescine-MAO-B pathway [102]. In this way, reactive astrocytes start to secrete GABA thus increasing inhibition, likely to be a defensive response against neuronal hyperexcitability that seems to be a universal result of AD progression [103,104]. Increase in MAO-B expression in astrocytes, which accompanies AD, also results in a hyperproduction of hydrogen peroxide that may instigate neuronal damage and death [105].

Metabolic deficits [106] and mitochondrial dysfunction also contribute to AD progression [107,108]. Extensive transcriptomics and proteomics studies revealed deficient mitochondrial bioenergetics in the AD brains [109,110,111]. Mitochondrial genes are altered in astrocytes from AD human brains as we discussed in detail in next section [112,113]. Exposure of mouse astrocytes to β-amyloid up-regulates superoxide dismutase thus increasing oxidative stress [114]; while continuous infusion of β-amyloid into mice brains results in substantial increase in production of hydrogen peroxide [115]. H_2_O_2_ overproducing astrocytes have been recently detected in the brains of AD model mice [105]. The β-amyloid/APP present in the mitochondrial inner membrane impairs the activity of Complex IV [116]. The toxic effect of β-amyloid on astrocytes is manifested by mitochondrial depolarisation with subsequent loss of Ca^2+^ homeostasis [117]. Astrocytic mitochondria are concentrated near the sites of homeostatic transport [118,119] providing energy of Na^+^/K^+^-ATPase that in turn drives accumulation of neurotransmitters, of which glutamate is the most prominent; deficits in ATP supply may affect glutamate clearance and promote excitotoxicity. Mitochondrial dynamics and function is also impaired in human astrocytes bearing APOE4 allele [120]. Another fundamental neuroprotective capability of astrocytes is linked to mitochondrial quality control and mitochondrial degradation. Astrocytes assist neuronal mitochondrial recycling through transmitophagy, when crippled neuronal mitochondria are transported to astrocytes where they undergo degradation [121]. Moreover, there are some indications that astrocytic mitochondria can be shuttled into neurones and assist to neuronal bioenergetics; in particular these processes seem to support neuroprotection after stroke [122]. Neuronal-astrocytic transmitophagy has been found to exert neuroprotection in the context of Parkinson disease [123]; whether this process contributes to the AD remains an exciting and albeit unanswered question.

Astrocytes potentially contribute to neuronal damage in other human neurodegenerative diseases such as Parkinson’s disease [124,125,126], Huntington’s disease [127,128], multiple sclerosis [129,130] and amyotrophic lateral sclerosis [131], indicating a direct contribution of these cells to a general programme of neurodegeneration. Most probably, astrocyte states and phenotypes differ among diseases, and even at different stages of a specific disease; further analyses are needed to dissect specific molecular pathways related to specific disease stages.

At the same time, astrocytes can exert neuroprotection at different stages of AD. Both astrogliosis and microgliosis in response to β-amyloid increase glial secretion of transforming growth factor β (TGF-β) (Figure 3). TGF-β protects neurones from β-amyloid toxicity and enhances β-amyloid clearance by microglia [88,99]. Moreover, astrocytes surrounding β-amyloid plaques demonstrate phagocytic activity and are able to phagocytose neuritic dystrophies in both mouse models and AD patients’ brains, further suggesting beneficial roles of astrocytes in AD [132]).

These data show that astrocytes actively contribute to the pathogenesis of AD. At the same time many questions remain to be addressed. What astroglial states/phenotypes are found at different stages of AD? How do astrocyte states/phenotypes differ between brain regions, which are known to have different vulnerability to AD? How do astrocytes crosstalk with other brain cells? Are they able to promote neurodegeneration? How do AD risk genes modulate astroglia responses in AD? New methodologies such as RNA sequencing and spatial transcriptomics in combination with the use of human iPSC-derived models and CRISPR-based studies are providing deeper understanding into how astrocytes evolve during the course of AD.

### 3.2. Astrocyte Genes and Altered Molecular Pathways in AD

RNA sequencing approaches are providing novel information about astrocyte states and soon we will be able to relate these states to different stages of AD. RNA sequencing analyses on acutely isolated mouse astrocytes revealed increased expression of inflammatory response genes (*Cst7*, *Ccl4*, *Il1b*, *Clec7a*, *Tyrobp)* and reduced expression of neuronal support genes (*Hes5)* and cholesterol biosynthesis genes (*Tm7sf2, Cyp51, Mvd*) in astrocytes from AD model mice (APPswe/PS1dE9) compared to healthy controls [133] (Table 1). When looking at specific genes, mouse astrocytes upregulate *Gfap, Bcl3, Serpina3n, Cyb5r2, Chil4, Bdkrb2, Rnase4* and the complement cascade genes *C4a, C4b* in AD model mice (PS2APP and APP/PS1) compared to control mice [134,135] (Table 1). In AD and healthy human post-mortem brains, transcriptional analyses of isolated astrocytes from different regions revealed differential expression of genes in pathways regulating cytoskeleton (*MYO6, KIF21A, ACTNB*), cell signalling (*IGF1R, PIK3R1, MAP3K12*), tight junctions (*GJC1, ZO1, TJAP1*) [136], and lipid metabolism (*ACOT1, ACOT2*) [137], as well as dysregulation of mitochondria-related genes (*TRMT61B, FASTKD2, NDUFA4L2*) and immune response genes (*CLU, C3, CD74*) [112] (Table 1). Overall, these data support astrocyte-specific contributions to AD mainly related to lipid metabolism, cholesterol biosynthesis, immune responses, and neuronal support, highlighting the importance of astrocyte activity in the neurodegenerative process.

While RNA sequencing of pooled astrocytes robustly corroborates the contribution of these cells to AD pathophysiology, it only captures expression of genes in grouped cells thus yielding population averages. Such transcriptome analyses can be affected by alterations in cell type composition of diseased vs. control samples and is unable to detect specific cell states, or changes in gene expression that occur in cell subsets. Therefore, single-cell or single-nuclei RNA sequencing are providing deeper insight in how cellular states evolve during AD progression.

Single-nucleus RNA sequencing of mouse astrocytes identified sub-populations of GFAP-low and GFAP-high astrocytes in both WT and AD mice (5xFAD); in addition, a unique cluster of disease-associated astrocytes (DAA) was detected in the AD mice [138]. The DAA cluster was enriched in *Gfap, Serpina3n*, *Ctsb, Apoe* and *Clu* among other genes (Table 1). While *Apoe* and *Clu* are known AD risk genes involved in amyloid processing, *Ctsb* encodes a lysosomal protease, Cathepsin B, linked to proteolytic processing of the amyloid precursor protein (APP), and *Serpina3n* encodes a protease inhibitor associated with increased β-amyloid accumulation. *Serpina3n* has also been identified in astrocytes from other AD model mice [134,135]; thus becoming a prime candidate for future investigations. Most of the detrimental astrocytic signature genes described in previous studies [101] are expressed by DAAs. Moreover, there are up to 18 genes shared by DAAs and disease-associated microglia [142], including *Apoe, Ctsb, Ctsd* and *Ctsl,* all encoding proteins involved in AD pathogenesis suggesting a general transcriptional program shared across cell types in AD. DAAs appear at early stages of AD and become more abundant as disease progresses suggesting that they not only respond to disease but also modulate disease course. Similar “pathological” astrocytes also emerge in aged WT mice and in ageing human brains [138], suggesting such molecular signatures are at least partially linked to age-related factors.

Single-nucleus RNA sequencing performed in parallel in both human control and AD brain samples and WT and AD mouse models (5xFAD) revealed remarkably different signatures between human and mice in astrocytes, as well as in microglia and oligodendrocytes [139]. While in AD mice astrocytes upregulate *Gfap* and *C4b*, in human AD brains astrocytes upregulate genes involved in extracellular matrix pathways including *NCAN* and *COL5A3* and downregulate genes involved in lipid and oxidative metabolism including *FABP5, HILPDA* and *SOD2* (Table 1) [139]. These data highlight the importance of analysing human samples to dissect molecular pathways involved in AD; direct translation from animal models could often be misleading.

Single-nucleus RNA sequencing of entorhinal cortex from human healthy and AD brains (*n* = 6 per group) revealed changes in specific astrocyte subpopulations [113]. While the AD astrocyte subcluster called a1 in this study upregulated genes involved in ribosomal, mitochondrial, neurone differentiation and heat shock responses, the AD astrocyte subcluster called a2 downregulated these processes and upregulated genes involved in transforming growth factor β (TGF-β) signalling and immune responses (Table 1). Upregulation of *C3* was also observed in AD astrocytes from the a2 subcluster in agreement with previous bulk RNA-seq analyses [112]. When analysing the expression of 1,000 GWAS candidate genes for AD and AD-related traits, *ADAMTS18, KCNN3* and *BIN1* were found upregulated, whereas *RGS20*, *FRMD4A* and *APOE* were downregulated in AD astrocytes (Table 1). *APOE* was downregulated in both a1 and a2 subclusters, in agreement with previous observations in human iPSC-derived astrocytes [94], while it was upregulated in microglial AD subcluster. The transcription factor *TFEB,* a master regulator of lysosomal function, is upregulated in AD astrocytes; *TFEB* was found to drive a network of ten AD GWAS genes (*BIN1, CLDN11, POLN, STK32B, EDIL3, AKAP12, HECW1, WDR5, LEMD2,* and *DLC1*). All these genes were dysregulated in AD astrocytes, suggesting that this master regulator controls the transition of astrocytes to a specific state identified by authors as “diseased” [113]. Single-nucleus sequencing was also performed in the prefrontal cortex of a bigger cohort of human control and AD brains (*n* = 24 per group) and confirmed *APOE* downregulation in AD astrocytes along with upregulation in microglia [140]. Subclustering of astrocyte nuclei revealed four subpopulations of cells with one subcluster called Ast1 enriched with AD cells that upregulated *GLUL* and the AD risk gene *CLU* [140] (Table 1), previously found upregulated in reactive astrocytes in response to neurodegeneration [143]. Recent single-nucleus sequencing of the entorhinal cortex and the superior frontal gyrus from human healthy brains (*n* = 3), early (*n* = 4) and advanced (n = 3) stages of AD also revealed an astrocyte subpopulation expressing higher levels of GFAP, called GFAP-high [141]. GFAP-high astrocytes upregulate *CD44* and *TNC*, both involved in interactions with the extracellular matrix; as well as *HSPB1* and *HSP90AA1*, chaperones involved in proteostasis. Interestingly, GFAP-high astrocytes downregulated genes involved in glutamate and GABA homeostasis (*SLC1A2, SLC1A3, GLUL* and *SLC6A11*) and synaptic adhesion/maintenance (*NRXN1, CADM2, PTN* and *GPC5*), indicating they may have compromised homeostatic function [141] (Table 1).

Overall, these studies provide complementary snapshots of astrocytic responses to pathology in the AD brain. Although there is still an acute need for more in-depth RNA sequencing analyses combined with large-scale meta-analyses on astrocyte transcriptomic datasets [144], the identification of genes and transcription factors that orchestrate the conversion of control to AD-associated astrocytes can already pinpoint specific molecular processes. In the coming years, integration of the most advanced sequencing technologies i.e., spatial transcriptomics [145,146] with multi-omics approaches i.e., epigenomics, proteomics and metabolomics [147,148,149] will allow validation of the present findings and provide specific mechanisms for therapeutic intervention.

## 4. Conclusions and Future Directions

Astrocytes have multiple functions in the brain and are essential for protection of neurones and maintenance of homoeostasis. However, under different pathological conditions including AD, they acquire diverse states, associated with either gain or loss of function contributing to neuroinflammation and neurodegeneration (Figure 3). A complete description of these cellular states, including multi-omics approaches combined with morphological and functional analyses, will advance understanding of how astrocytes evolve in pathology and in the near future, we may be able to relate different astroglial states to specific stages of AD, which might lead to novel biomarkers and targets for therapeutic intervention.

## Figures and Tables

**Table 1:** Summary of differentially expressed genes (DEGs) and molecular pathways based on RNA sequencing analysis of astrocytes in Alzheimer’s disease. DEGs are shown comparing AD vs. control mice and AD patient vs. human healthy brain samples. Upregulated genes are shown in red, downregulated genes in blue and dysregulated genes in green.

Species	Brain Region	RNA-Seq Technique	Isolation Method	DEGs	Pathways	Refs
Mouse APP/PS1	Cortex	Bulk RNA-seq	GLT-1	*Cst7* *Ccl4* *Il1b* *Clec7a* *Tyrob* *Hes5* *Tm7sf2* *Cyp51* *Mvd*	Inflammatory response; Neuronal support; Cholesterol biosynthesis	[133]
Mouse PS2APP	Cortex	Bulk RNA-seq	GFAP	*Gfap* *Bcl3* *Serpina3n* *C4a* *C4b*		[135]
Mouse APP/PS1	Whole brain	Bulk RNA-seq	ACSA2	*Cyb5r2* *Chil4* *Bdkrb2* *Rnase4* *C4b*		[134]
Human	Lateral temporal cortex	Microarray	GFAP	*MYO6* *KIF21A* *ACTNB* *IGF1R* *PIK3R1* *MAP3K12* *GJC1* *ZO1* *TJAP1*	Cytoskeleton; Cell signalling; Cell junctions	[136]
Human	Parietal cortex	Bulk RNA-seq	unbiased	*ACOT1* *ACOT2*	Lipid metabolism	[137]
Human	Posterior cingulate cortex	Bulk RNA-seq	ALDH1L1	*TRMT61B* *FASTKD2* *NDUFA4L2* *CLU* *C3* *CD74*	Mitochondria; Immune response	[112]
Mouse 5XFAD	Hippocampus	Single-nuclei	unbiased	*Gfap* *Serpina3n* *Apoe* *Clu* *Ctsb* *Ctsd* *Ctsl*	Disease associated astrocytes (DAA) cluster	[138]
Mouse 5XFAD	Cortex	Single-nuclei	unbiased	*Gfap* *C4b*		[139]
Human	Prefrontal cortex	Single-nuclei	unbiased	*NCAN* *COL5A3* *FABP5* *HILPDA* *SOD2*	Extracellular matrix; Lipid and oxidative metabolism	[139]
Human	Entorhinal cortex	Single-nuclei	unbiased	*C3* *ADAMTS18* *KCNN3* *BIN1* *TFEB* *RGS20* *FRMD4A* *APOE* *CLDN1* *POLN* *STK32B* *EDIL3* *AKAP12* *HECW1* *WDR5* *LEMD2* *DLC1*	Ribosomal function; Mitochondrial function; Neurone differentiation; Heat shock responses; TGFβ signalling; Immune response	[113]
Human	Prefrontal cortex	Single-nuclei	unbiased	*GLUL* *CLU* *APOE*		[140]
Human	Entorhinal cortex; Superior frontal gyrus	Single-nuclei	unbiased	*CD44* *TNC* *HSPB1* *HSP90AA1* *SLC1A2* *SLC1A3* *GLUL* *SLC6A11* *NRXN1* *CADM2* *PTN* *GPC5*	Extracellular matrix interactions; Proteostasis; Glutamate/GABA homeostasis; Synaptic adhesion/maintenance	[141]
